# Dietary Polyphenols Curcumin and Resveratrol Exert Selective Anticancer Effects in Melanoma Cells

**DOI:** 10.3390/nu18030548

**Published:** 2026-02-06

**Authors:** Moon-Kyun Cho, Yeji Lee, Ki Dam Kim, Min Hyuk Choi, Sang-Han Lee, Dongsic Choi, Hae-Seon Nam, Yoon-Jin Lee

**Affiliations:** 1 Division of Molecular Cancer Research, Soonchunhyang Medical Research Institute, Soonchunhyang University, Cheonan 31511, Republic of Korea; mkcho@schmc.ac.kr (M.-K.C.); m1037624@sch.ac.kr (S.-H.L.); namhs@sch.ac.kr (H.-S.N.); 2 Department of Dermatology, Soonchunhyang University Hospital, Seoul 04401, Republic of Korea; 3 Department of Biochemistry, College of Medicine, Soonchunhyang University, Cheonan 31511, Republic of Korea; yjyjlee37@naver.com (Y.L.); dongsicchoi@gmail.com (D.C.); 4 Department of Tropical Medicine, College of Medicine, Soonchunhyang University, Cheonan 31511, Republic of Korea

**Keywords:** melanoma, curcumin, resveratrol, polyphenols, combination therapy, apoptosis, cell cycle arrest, ATP depletion, signaling pathways

## Abstract

**Background/Objectives:** Although curcumin (CUR) and resveratrol (RSV) are natural polyphenolic compounds with reported anticancer and anti-inflammatory properties, their combined anticancer effects in melanoma cells remain incompletely characterized. This study aimed to evaluate the anticancer efficacy of CUR and RSV, individually, and in combination, in melanoma cells compared to normal melanocytes. **Methods:** Cell viability and intracellular ATP levels were quantified, and dose–response analyses performed. Cellular morphology and nuclear alterations were examined by phase-contrast microscopy and DAPI staining. Cell cycle distribution and apoptosis were analyzed by Muse™ Cell Analyzer with dedicated assay kits. Survival- and death-related signaling proteins were evaluated by Western blotting. **Results:** Combined treatment with CUR (60 μM) and RSV (40 μM) for 48 h synergistically reduced melanoma cell viability and markedly depleted intracellular ATP levels, while exerting minimal cytotoxic effects on normal melanocytes. CUR/RSV co-treatment induced pronounced morphological and nuclear alterations, significantly increased apoptotic cell populations, and modulated key signaling pathways regulating cell survival and programmed cell death in melanoma cells. **Conclusions:** These findings demonstrate that combined CUR and RSV treatment exerts enhanced, melanoma-selective anticancer activity while sparing normal melanocytes. The results provide a strong experimental rationale for further in vivo validation of CUR/RSV-based combination strategies as a potential therapeutic approach for melanoma.

## 1. Introduction

Melanoma, an aggressive tumor arising from melanocytes, accounts for the highest mortality among skin cancers, despite representing a small proportion of cases [1,2]. Its marked metastatic capacity and increasing global incidence underscore a persistent clinical need for improved therapeutic strategies [1]. Although surgery can effectively remove early-stage melanoma, limited treatment responsiveness means that long-term survival in metastatic disease remains poor [2]. The tumor microenvironment is further characterized by immune suppression, metabolic reprogramming, and resistance to programmed cell death, all of which contribute to disease progression and treatment failure.

Recent therapeutic advances have centered on targeted MAPK pathway inhibition and immune checkpoint blockade, including antibodies against PD-1 and CTLA-4, which have shown notable clinical benefit in subsets of patients [3,4,5]. BRAF and MEK inhibitors induce rapid tumor regression, whereas immunotherapies can yield durable responses. However, therapeutic resistance, immune-related toxicities, and disease relapse remain common limitations [3,4,5]. Metabolic plasticity and compensatory signaling frequently allow melanoma cells to escape targeted inhibition, highlighting the unmet need for complementary strategies that can enhance anticancer efficacy while minimizing toxicity.

Natural polyphenolic compounds have therefore become attractive candidates, due to their pleiotropic biological activities and favorable safety profiles [6,7]. Unlike single-target pharmacological agents, polyphenols can modulate multiple pathways associated with proliferation, survival, oxidative stress, inflammation, and programmed cell death—properties relevant to the molecular heterogeneity of melanoma [3]. Importantly, many polyphenols are dietary constituents and are investigated within the context of nutritional oncology and phytochemical-based cancer prevention, increasing interest in their translational potential as adjunctive agents that can potentiate chemotherapy or targeted therapy responses, while mitigating adverse effects.

**Figure 1:**
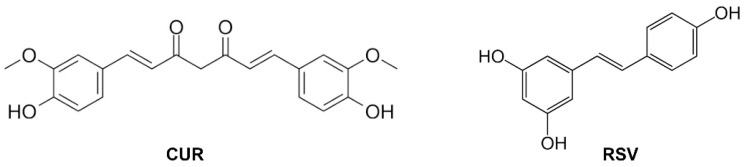
The chemical structures of two dietary polyphenols, curcumin (CUR) and resveratrol (RSV). These bioactive compounds offer well-established antioxidant and anti-inflammatory activities, and were utilized in this study to evaluate their combined anti-cancer effects on melanoma cells.

Curcumin (CUR) and resveratrol (RSV) are among the most extensively studied dietary polyphenols with reported anticancer activities. Their chemical structures (Figure 1) correspond to a diarylheptanoid (CUR) and a stilbene derivative (RSV), respectively, and are associated with antioxidant, anti-inflammatory, and anticancer properties.

CUR suppresses melanoma growth by inducing cell cycle arrest and apoptosis, and by inhibiting NF-κB, PI3K/AKT, and MAPK signaling [8,9,10]. RSV exhibits similar activities and also modulates angiogenesis and metastatic processes [11,12,13]. However, when administered individually, relatively high concentrations for efficacy are often required, which may limit translational value.

Importantly, CUR and RSV exhibit partially overlapping yet distinct molecular targets, suggesting that their combination may cooperatively disrupt key survival and metabolic pathways that underlie melanoma progression and therapy resistance. In particular, simultaneous modulation of PI3K/AKT- and MAPK/ERK1/2-associated signaling, together with redox and inflammatory pathways, provides a mechanistic rationale for combining CUR and RSV to enhance anticancer efficacy while reducing the required dose of each compound.

Combination therapy using natural compounds has been proposed to reduce required doses while enhancing efficacy through the cooperative modulation of survival pathways [14]. In melanoma, aberrant PI3K/AKT and MAPK/ERK1/2 signaling contribute to proliferation, therapeutic resistance, and immune evasion [3,15], and resistance extends beyond apoptosis to necroptosis and other non-apoptotic death pathways [15]. Such multilayered resistance underscores the potential value of phytochemical combination strategies. Because CUR and RSV are bioactive dietary constituents with favorable safety profiles, identifying synergistic or additive interactions between them may provide nutritionally relevant anticancer strategies with reduced toxicity burdens, compared to conventional treatments.

To address these gaps, this study systematically examined the effects of CUR and RSV, alone and in combination, on human melanoma G361 cells and normal human epidermal melanocytes (HEMn-MP). Through complementary metabolic, morphological, and molecular assays, we evaluated viability, ATP depletion, cell cycle dynamics, apoptosis induction, and modulation of survival- and death-associated signaling pathways. Collectively, these findings support CUR/RSV co-treatment as a complementary polyphenol-based approach in melanoma research and highlight the nutritional relevance of dietary phytochemicals as potential anticancer strategies.

## 2. Materials and Methods

### 2.1. Reagents and Antibodies

Curcumin (CUR) and resveratrol (RSV) were purchased from Sigma-Aldrich (St. Louis, MO, USA), and dissolved in dimethyl sulfoxide (DMSO) to prepare concentrated stock solutions. Additional reagents, including MTT, DAPI, paraformaldehyde, and β-actin antibody, were obtained from the same supplier. Antibodies recognizing cleaved caspase-3, cleaved PARP, phospho- and total AKT and ERK1/2, RIP, and MLKL, as well as horseradish peroxidase (HRP)-conjugated secondary antibodies, were purchased from Cell Signaling Technology (Danvers, MA, USA). Prior to immunoblotting, primary and secondary antibodies were diluted in a casein-based blocking buffer (Thermo Fisher Scientific, Waltham, MA, USA).

### 2.2. Cell Maintenance

Normal human epidermal melanocytes (HEMn-MP; Cascade Biologics, Portland, OR, USA) were maintained in Medium 254 supplemented with Human Melanocyte Growth Supplement, according to the supplier’s instructions. Human melanoma G361 cells (ATCC, Manassas, VA, USA) were cultured in Dulbecco’s Modified Eagle Medium (WelGene, Gyeongsan, Republic of Korea) containing 5% fetal bovine serum (Gibco, Gaithersburg, MD, USA), 100 U/mL penicillin, and 100 μg/mL streptomycin. All cells were incubated at 37 °C in a humidified atmosphere with 5% CO_2_.

### 2.3. Drug Exposure Conditions

Cells were treated with CUR (60 μM), RSV (40 μM), or a combination of both compounds for 48 h. Control cells received an equivalent volume of DMSO, which did not exceed 0.1% (*v*/*v*) of the total culture volume.

### 2.4. Cell Viability Assay

Cell viability was assessed by MTT colorimetric assay. Cells were seeded into 96-well plates and treated as indicated. Following incubation, MTT reagent was added, and formazan crystals were allowed to form at 37 °C. Crystals were solubilized with DMSO, and absorbance was measured at 550 nm using a GloMax-Multi Detection System (Promega, Madison, WI, USA).

### 2.5. Combination Effect Analysis

The interaction between CUR and RSV was analyzed using the combination index (CI) method as previously described [16]. CI values were interpreted as follows: CI < 1.0, synergistic; CI = 1.0, additive; CI > 1.0, antagonistic.

### 2.6. Intracellular ATP Measurement

Intracellular ATP content was quantified using the CellTiter-Glo^®^ Luminescent Cell Viability Assay (Promega, Madison, WI, USA). After drug exposure, assay reagent was added to equilibrated plates, and luminescence was recorded using a microplate reader. Data were normalized to untreated controls.

### 2.7. Morphological and Nuclear Evaluation

Changes in cell morphology were observed by phase-contrast microscopy. For nuclear visualization, cells were fixed with paraformaldehyde and stained with DAPI. Fluorescence images were obtained via Leica microscopy (Leica Microsystems GmbH, Wetzlar, Germany).

### 2.8. Cell Cycle Analysis

Cell cycle distribution was assessed using the Muse™ Cell Cycle Kit (Luminex Corporation, Austin, TX, USA). Ethanol-fixed cells were stained according to the manufacturer’s instructions, and DNA content was measured using a Muse™ Cell Analyzer (Merck Millipore, Darmstadt, Germany).

### 2.9. Apoptosis Quantification

Apoptotic populations were evaluated using the Muse™ Annexin V & Dead Cell Assay Kit (cat. no. MCH100105; Merck KGaA, Darmstadt, Germany). Stained cells were analyzed with a Muse™ Cell Analyzer to distinguish viable, early apoptotic, late apoptotic, and dead cells.

### 2.10. Immunoblotting

Whole-cell lysates were prepared using RIPA buffer, and protein concentrations were determined by bicinchoninic acid assay (Thermo Fisher Scientific). Equal amounts of protein were subjected to SDS-PAGE and transferred to PVDF membranes (Cytiva Life Sciences, Marlborough, MA, USA). Membranes were probed with primary antibodies overnight, followed by incubation with HRP-conjugated secondary antibodies (Table 1). Immunoreactive bands were visualized using enhanced chemiluminescence reagents and quantified using ImageJ software version 1.0 (National Institutes of Health, Bethesda, MD, USA). Molecular weight markers (kDa) were included and are indicated in the corresponding figures. Uncropped and high-resolution immunoblot images are provided in the Supplementary Materials.

### 2.11. Statistical Analysis

All experiments were performed using at least three independent biological replicates conducted on separate days (*n* = 3). Experimental data are expressed as the mean ± standard deviation (SD). Statistical significance was assessed by one-way analysis of variance (ANOVA), followed by Tukey’s multiple comparison test, using GraphPad Prism software (version 9.5.1). Exact *p*-values for all quantitative analyses are provided in the Supplementary Materials.

## 3. Results

### 3.1. CUR and RSV Selectively Reduce Viability and ATP Production in Melanoma Cells

**Figure 2:**
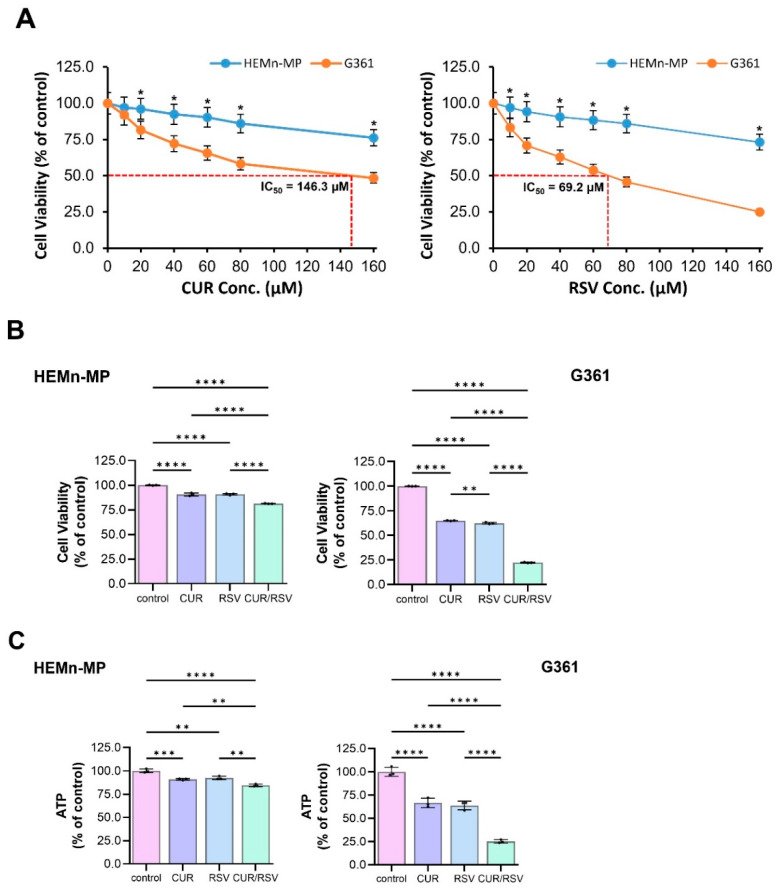
Effects of curcumin (CUR) and resveratrol (RSV) on cell viability and intracellular ATP levels in melanoma G361 cells and normal human melanocytes. (**A**) Dose–response curves showing the effects of CUR (**left**) and RSV (**right**) on cell viability in G361 and HEMn-MP cells. IC_50_ values for CUR and RSV were calculated for G361 cells using nonlinear regression. (**B**) Single-dose treatment (CUR, 60 μM; RSV, 40 μM; 48 h) reduced viability in G361 cells with relatively limited effects in HEMn-MP cells; CUR/RSV co-treatment further potentiated growth inhibition in G361 cells. (**C**) CUR/RSV co-treatment markedly decreased intracellular ATP levels in G361 cells, but not in HEMn-MP cells. Data are presented as the mean ± SD from ≥3 independent experiments. Statistical significance was assessed by one-way ANOVA with Tukey’s post hoc test (* *p* < 0.05, ** *p* < 0.01, *** *p* < 0.001, **** *p* < 0.0001 vs. control).

Dose–response analysis showed that CUR and RSV reduced melanoma cell viability in a concentration-dependent manner, while exerting comparatively limited cytotoxicity in normal melanocytes (Figure 2A). Following CUR treatment at (10, 20, 40, 60, 80, and 160) μM, HEMn-MP cells maintained (96.9, 96.0, 92.3, 90.2, 85.9, and 76.0)% viability, whereas G361 cells showed lower viability of (91.9, 81.6, 72.1, 65.5, 58.2, and 48.3)%, respectively. A similar pattern was observed for RSV: HEMn-MP cells at (10, 20, 40, 60, 80, and 160) μM retained (96.9, 94.2, 90.5, 88.3, 85.9, and 73.1)% viability, while G361 cells decreased to (83.1, 70.8, 62.8, 53.7, 45.6, and 25.0)%, respectively. Based on these data, 60 μM CUR and 40 μM RSV were selected as the highest concentrations that were non-cytotoxic to HEMn-MP cells (>90% viability), while producing measurable inhibition in melanoma cells.

Under these conditions (Figure 2B), CUR and RSV alone yielded 90.54% and 90.84% viability in HEMn-MP cells, respectively, and 64.99% and 62.37% viability in G361 cells, respectively. Notably, CUR/RSV co-treatment further reduced G361 viability to 22.30%, whereas HEMn-MP viability remained relatively stable at 81.29%, indicating preferential cytotoxicity toward melanoma cells. Drug interaction analysis using the combination index (CI) method showed minimal interaction in HEMn-MP cells (CI = 1.068) and strong synergy in G361 cells (CI = 0.435), supporting cancer-selective synergy.

To assess metabolic consequences, intracellular ATP levels were quantified (Figure 2C). In HEMn-MP cells, ATP content was largely maintained, with CUR, RSV, and CUR/RSV showing (90.88, 92.44, and 84.40)% vs. control, respectively, while in contrast, G361 cells exhibited pronounced ATP depletion, with CUR, RSV, and CUR/RSV showing (66.50, 63.81, and 25.10)%, respectively. Although these data demonstrate a strong association between CUR/RSV co-treatment and ATP depletion in melanoma cells, the temporal relationship between ATP loss and downstream signaling or cell death execution was not directly assessed. Exact *p*-values for all quantitative comparisons are provided in the Supplementary Materials. Collectively, these results indicate that CUR and RSV cooperatively suppress melanoma cell viability and metabolic activity, while exerting comparatively limited effects on normal melanocytes.

### 3.2. CUR + RSV Induce Morphological Changes and Nuclear Condensation in Melanoma Cells

**Figure 3:**
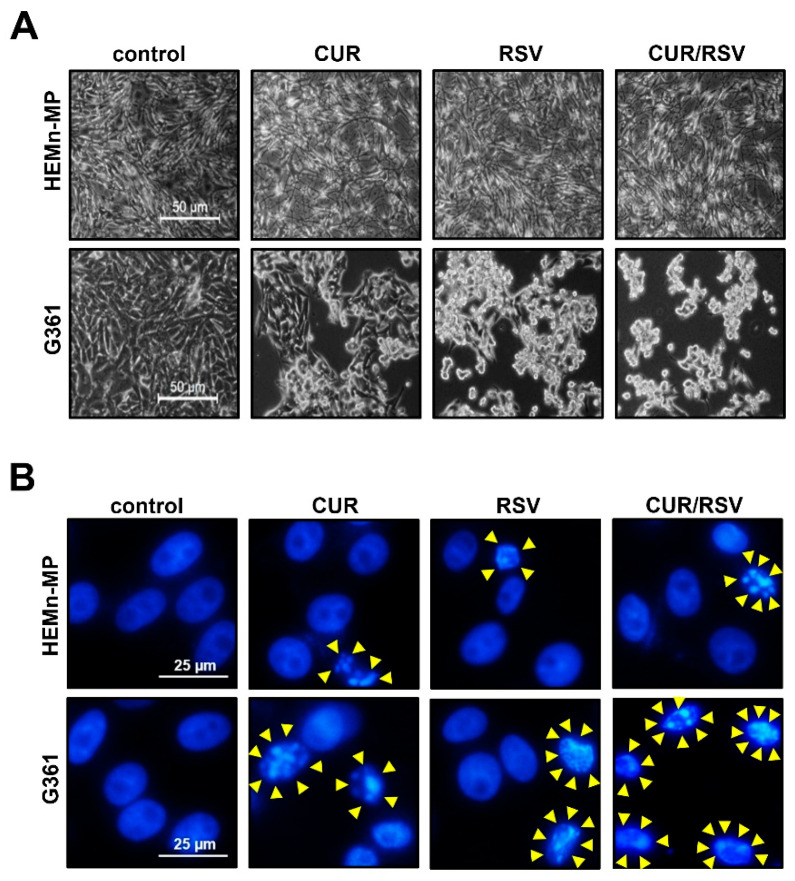
Effects of curcumin (CUR) and resveratrol (RSV) on cellular morphology and nuclear integrity in normal melanocytes and melanoma cells. (**A**) Representative phase-contrast images of HEMn-MP and G361 cells after 48 h exposure to CUR (60 μM), RSV (40 μM), or CUR/RSV. HEMn-MP cells maintained spindle-like morphology, whereas G361 cells displayed progressive shrinkage and detachment, particularly under CUR/RSV co-treatment. Scale bar = 50 μm. (**B**) DAPI staining shows nuclear condensation and fragmentation (yellow arrowheads) in G361 cells following CUR/RSV co-treatment. Scale bar = 25 μm.

Morphological assessment revealed distinct cellular responses to CUR and RSV (Figure 3). Phase-contrast microscopy showed that G361 cells exhibited cell shrinkage, membrane blebbing, and reduced cell density following single-agent exposure, with the most pronounced alterations observed under co-treatment. In contrast, HEMn-MP cells largely retained spindle-like morphology across all conditions. In G361 cells, DAPI staining further demonstrated nuclear condensation and fragmentation after CUR/RSV treatment, consistent with apoptotic nuclear morphology, whereas in HEMn-MP cells, no appreciable nuclear alterations were detected. These findings indicate that CUR and RSV cooperatively induce cytomorphological hallmarks of cell death preferentially in melanoma cells.

### 3.3. CUR/RSV Induce G0/G1 Arrest and Apoptosis in Melanoma Cells

**Figure 4:**
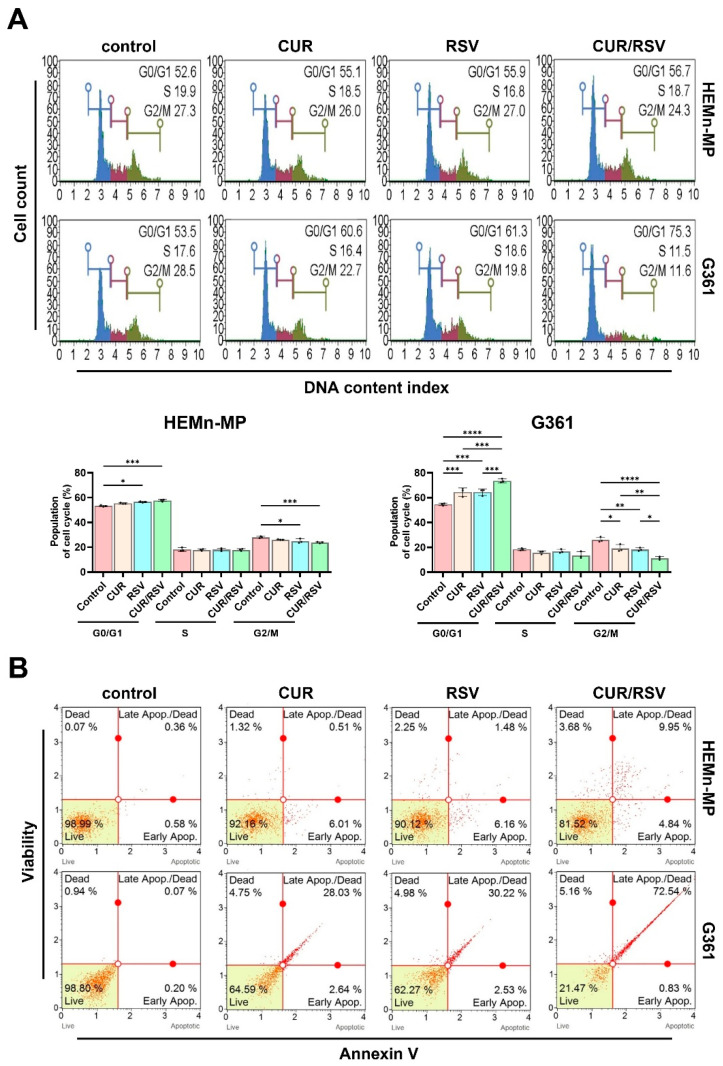
Effects of curcumin (CUR) and resveratrol (RSV) on cell cycle distribution and apoptosis in normal melanocytes and melanoma cells. (**A**) After 48 h exposure to CUR (60 μM), RSV (40 μM), or CUR/RSV, cell cycle distribution in HEMn-MP and G361 cells was quantified using a Muse™ Cell Analyzer. The colored lines represent different cell cycle phases: G0/G1 (blue), S phase (red), and G2/M (green). CUR/RSV co-treatment increased G0/G1 accumulation in G361 cells compared with single agents, while HEMn-MP cells showed minimal changes. Data are the mean ± SD (*n* = 3). (**B**) Apoptotic populations were quantified using the Muse™ Annexin V & Dead Cell assay. CUR/RSV markedly increased early and late apoptosis in G361 cells. Statistical significance was assessed by one-way ANOVA with Tukey’s post hoc test (* *p* < 0.05; ** *p* < 0.01; *** *p* < 0.001; **** *p* < 0.0001).

Cell cycle distribution and apoptosis were quantified using a Muse™ Cell Analyzer with dedicated assay kits (Figure 4). In HEMn-MP cells, CUR and RSV, alone or in combination, produced minimal changes in cell cycle distribution. In the control, CUR, RSV, and CUR/RSV groups, the G0/G1 fraction was (53.23, 55.30, 56.47, and 57.67)%; S-phase fractions remained stable at (18.13, 17.90, 18.07, and 17.93)%, while G2/M fractions showed only modest decreases at (28.07, 26.17, 24.93, and 23.77)%, respectively.

In contrast, following CUR/RSV exposure, G361 melanoma cells exhibited pronounced cell cycle shifts. With CUR and RSV alone, the G0/G1 fraction increased from 54.63 (control) to 64.43 and 64.40)%, respectively, and with CUR/RSV, further to 73.57%. Concomitantly, in the control, CUR, RSV, and co-treatment groups, S–phase cells declined from (18.33 to 15.83, 16.83, and 13.63)%, while G2/M populations decreased from (26.13 to 19.23, 18.33, and 11.40)%, respectively, indicating a marked G0/G1 arrest accompanied by reduced DNA synthesis and mitotic activity.

Apoptosis was assessed using the Muse™ Annexin V & Dead Cell assay (Figure 4B). In HEMn-MP cells, following CUR, RSV, and CUR/RSV treatment, the combined fraction of early apoptotic, late apoptotic, and dead cells increased modestly from 1.9 (control) to 7.7, 8.8, and 18.1)% respectively. By contrast, G361 cells displayed a robust apoptotic response, with total apoptotic/dead fractions with CUR and RSV alone rising from 1.6 (control) to 34.5 and 37.9)%, and with CUR/RSV, further to 78.9%. Statistical significance for all comparisons is indicated in the figures, with exact *p*-values summarized in the Supplementary Materials. Collectively, these results indicate that CUR and RSV cooperatively induce G0/G1 cell cycle arrest and apoptosis preferentially in melanoma cells, supporting their selective anticancer activity.

### 3.4. CUR + RSV Modulate Apoptosis- and Survival-Associated Cell Signaling Pathways

**Figure 5:**
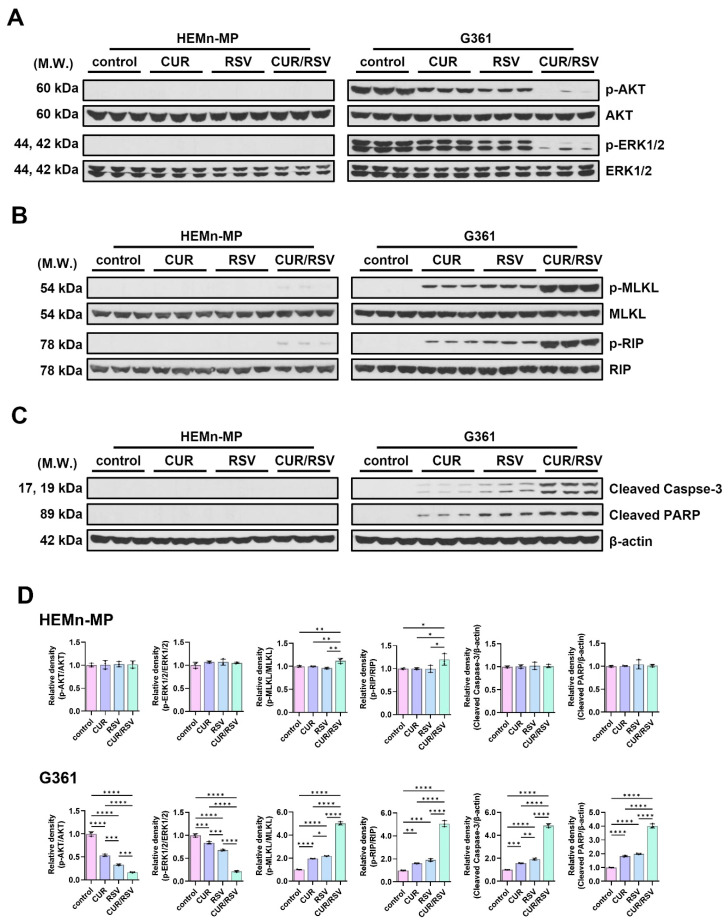
Effects of CUR/RSV on survival and cell death signaling in melanoma and normal melanocytes. (**A**) Representative Western blot images showing survival-related signaling proteins, including p-AKT/AKT and p-ERK1/2/ERK1/2, in HEMn-MP and G361 cells treated with CUR, RSV, or CUR/RSV. (**B**) Expression of necroptosis-associated markers p-RIP/RIP and p-MLKL/MLKL under the same treatment conditions. (**C**) Apoptotic markers, including cleaved caspase-3 and cleaved PARP, with β-actin used as a loading control. (**D**) Densitometric quantification of protein expression levels normalized to total protein or β-actin, as indicated. Molecular weight markers (kDa) are indicated to the left of each immunoblot. CUR/RSV co-treatment in G361 cells suppressed pro-survival signaling while enhancing necroptotic and apoptotic signaling pathways. In contrast, HEMn-MP cells exhibited minimal changes in survival- and death-related protein expression. Data are presented as the mean ± SD (*n* = 3). Statistical significance was assessed by one-way ANOVA with Tukey’s post hoc test (* *p* < 0.05; ** *p* < 0.01; *** *p* < 0.001; **** *p* < 0.0001 vs. control).

To elucidate the molecular basis of the selective anticancer activity of CUR and RSV, survival- and cell death-associated proteins were examined by Western blotting with densitometric quantification (Figure 5A–D). In normal HEMn-MP cells, phosphorylation of AKT and ERK1/2 was largely preserved across treatments. For CUR, RSV, and CUR/RSV, the p-AKT/AKT and p-ERK1/2/ERK1/2 ratios were (1.011 and 1.071), (1.023 and 1.069), and (1.017 and 1.050), respectively (Figure 5A). Consistently, necroptosis-related markers (p-MLKL/MLKL and p-RIP/RIP) remained minimally altered in HEMn-MP cells; under CUR/RSV co-treatment, p-MLKL/MLKL and p-RIP/RIP were (1.121 and 1.198), respectively (Figure 5B). Apoptotic markers, including cleaved caspase-3 and cleaved PARP, also showed negligible changes in normal melanocytes, with cleaved caspase-3/β-actin and cleaved PARP/β-actin values of (1.015 and 1.014) under CUR/RSV co-treatment, indicating minimal activation of programmed cell death signaling (Figure 5C). In contrast, in response to CUR/RSV, G361 melanoma cells exhibited pronounced pathway modulation. Co-treatment markedly suppressed survival signaling, with p-AKT/AKT reduced from 1.000 (control) to 0.163, and p-ERK1/2/ERK1/2 reduced from 1.000 to 0.216 (Figure 5A). Concurrently, necroptotic signaling was strongly induced, as reflected by increases in p-MLKL/MLKL from (1.000 to 5.017), and p-RIP/RIP from (1.000 to 5.060) (Figure 5B). Apoptotic signaling was also enhanced, with cleaved caspase-3/β-actin and cleaved PARP/β-actin increasing from (1.000 to 4.852 and 4.012), respectively (Figure 5C). Although single-agent treatments produced measurable effects, the magnitude of pathway modulation was consistently greater under co-treatment.

Densitometric analyses across all signaling markers confirm that CUR/RSV co-treatment selectively suppresses AKT/ERK1/2-mediated survival signaling while concomitantly activating apoptotic and necroptotic pathways in melanoma cells, with minimal effects in normal melanocytes (Figure 5D).

Collectively, these data demonstrate that CUR/RSV selectively inhibits AKT/ERK1/2 survival signaling while concomitantly activating apoptotic and necroptotic pathways in melanoma cells, with minimal effects in normal melanocytes. This coordinated suppression of pro-survival signaling and induction of programmed cell death provides mechanistic support for the cancer-selective synergistic activity of the CUR/RSV combination.

## 4. Discussion

In this study, we demonstrate that combined treatment with the dietary polyphenols curcumin (CUR) and resveratrol (RSV) exerts a selective and synergistic anticancer effect in human melanoma G361 cells, while sparing normal human epidermal melanocytes (HEMn-MP). Key findings include cooperative suppression of cell viability and ATP levels, induction of G0/G1 cell cycle arrest, and concomitant activation of apoptotic and necroptotic pathways, together with melanoma-selective inhibition of AKT and ERK1/2 signaling. Importantly, although these observations are derived from in vitro models, they provide mechanistic insights that can inform the design of subsequent in vivo and translational studies.

Dietary polyphenols have attracted attention as multi-target modulators of cancer-relevant processes, particularly when combinations are used to amplify efficacy while maintaining an acceptable safety margin. In this study, curcumin (CUR) and resveratrol (RSV) displayed a cooperative anticancer effect in human melanoma G361 cells, while exerting comparatively limited effects in normal human epidermal melanocytes (HEMn-MP). This differential response is consistent with nutritional oncology concepts that leverage pleiotropic bioactives to exploit tumor-specific dependencies, including elevated survival signaling and heightened metabolic demand [17,18,19,20]. Importantly, the interaction profile was supported by quantitative combination analysis, where CI values indicated synergy in melanoma cells but minimal interaction in normal melanocytes, strengthening the interpretation that the combination preferentially targets malignant cells, rather than causing nonspecific toxicity [17,18].

A principal finding is the selective reduction in melanoma cell viability accompanied by pronounced ATP depletion under CUR/RSV co-treatment. The energetic phenotype is noteworthy because ATP availability is a central determinant of cell fate under stress: when bioenergetic supply cannot meet demand, cells lose the capacity to maintain ion gradients, macromolecular synthesis, proteostasis, and checkpoint control, thereby increasing susceptibility to regulated cell death [19,20]. Melanoma cells often exhibit metabolic flexibility and increased basal energetic pressure, which, while providing resilience, also creates vulnerabilities when multiple metabolic and signaling constraints are simultaneously imposed [20]. Importantly, whether ATP depletion represents an initiating event or a downstream consequence of apoptosis and necroptosis execution remains to be clarified. From an in vivo perspective, these results suggest that melanoma cells may be particularly sensitive to interventions that concurrently disrupt metabolic homeostasis and survival signaling, a concept that warrants validation in animal models with intact metabolic and stromal contexts. The marked ATP decline observed in G361 cells after co-treatment, together with the comparatively preserved ATP levels in HEMn-MP, suggests that CUR/RSV imposes a level of metabolic stress that melanoma cells cannot efficiently buffer, whereas normal melanocytes maintain sufficient metabolic homeostasis. Prior studies have shown that RSV can influence glycolysis-associated programs and promote anti-proliferative effects in melanoma models, and curcumin has also been linked to mitochondrial stress and apoptosis in melanoma contexts, supporting the plausibility of a metabolism-death axis that, when both polyphenols are combined, is amplified [21,22,23,24].

In parallel with metabolic impairment, CUR/RSV produced a robust cell cycle effect in melanoma cells, characterized by an increased G0/G1 population and reduced S and G2/M fractions. Cell cycle arrest can contribute directly to growth inhibition, and also function as a sensitizing context for cell death, because under unfavorable energetic conditions, arrested cells may be less able to resolve stress, repair damage, or complete replication [25,26]. Such coordinated effects on metabolism and cell cycle regulation provide a mechanistic framework that may be exploited in vivo to suppress tumor growth while limiting toxicity to normal tissues. The G0/G1 accumulation in G361 cells was substantially greater with co-treatment than with either agent alone, consistent with the cooperative disruption of proliferative signaling and checkpoint control. Both CUR and RSV have been reported to modulate G1/S transition regulators and upstream kinase pathways across multiple cancer systems, and ERK1/2 pathway suppression in melanoma is well recognized to translate into G1 checkpoint reinforcement and reduced cyclin-driven progression [21,26,27]. The relative absence of meaningful cell cycle perturbation in HEMn-MP cells under identical exposures further supports the selectivity of the combination for malignant proliferative programs.

Programmed cell death was a major endpoint of CUR/RSV exposure in melanoma cells. Muse™ Annexin V/Dead Cell analysis indicated extensive apoptosis in G361 cells under co-treatment, whereas normal melanocytes exhibited comparatively limited apoptosis. Apoptosis resistance represents a central barrier in melanoma biology and therapy, often sustained by hyperactive MAPK/ERK1/2 and PI3K/AKT signaling [20,25]. Although apoptosis was clearly engaged, future studies will be required to determine whether pharmacological or genetic inhibition of necroptosis shifts cell death toward apoptosis or partially restores melanoma cell viability. In the current study, the concordant increase in cleaved caspase–3 and cleaved PARP in melanoma cells supports caspase-dependent execution as an important component of the cytotoxic response. Prior melanoma-focused reports have documented that curcumin can induce apoptosis with caspase activation and PARP cleavage, and can attenuate pro-survival signaling, consistent with the present pattern [22,23]. Resveratrol has also been shown to suppress melanoma proliferation and induce apoptosis in several experimental settings, including p53-associated mechanisms in certain contexts, supporting its capacity to lower the death threshold in melanoma cells [28,29,30]. While apoptosis readouts do not by themselves define upstream causality, the alignment of Annexin V positivity with caspase and PARP cleavage provides coherent evidence that, under co-treatment, apoptosis is strongly engaged. These apoptosis-associated responses represent measurable endpoints that could be readily assessed in future in vivo melanoma models.

A distinctive mechanistic aspect of the present results is the concurrent activation of necroptosis-associated signaling in melanoma cells, evidenced by the increased phosphorylation of RIP and MLKL under CUR/RSV co-treatment. Necroptosis is a regulated cell death program mediated by RIPK−MLKL signaling [31]. Because necroptosis can influence inflammatory signaling, DAMP release, and immune cell recruitment, these findings raise the possibility that CUR/RSV-induced necroptotic signaling may have immune-relevant consequences in vivo, particularly in immunocompetent melanoma models [32,33]. Comprehensive mechanistic reviews emphasize that MLKL phosphorylation is a key biochemical marker of necroptosis execution, although regulation can be context dependent, while functional validation is recommended to confirm pathway contribution [34,35]. The simultaneous observation of apoptotic markers (cleaved caspase-3/PARP) and necroptotic markers (p-RIP/p-MLKL) suggests that rather than relying on a single route, CUR/RSV engages multiple regulated death programs to eliminate melanoma cells. Such multi-pathway engagement could be advantageous in melanoma, where heterogeneity and adaptive resistance can allow subsets of cells to evade a single death mechanism [20]. At the signaling level, the melanoma-selective suppression of phosphorylated AKT and ERK1/2 provides a plausible upstream basis for the combined metabolic, cell cycle, and death phenotypes. PI3K/AKT and MAPK/ERK1/2 are central survival hubs that regulate proliferation, metabolism, and apoptosis thresholds, and their dysregulation is strongly linked to melanoma progression and therapy resistance [20,25,36]. Reviews of PI3K/AKT-targeted therapy emphasize the centrality of this pathway and the challenges posed by feedback loops and redundancy, particularly when single-node inhibition permits compensatory signaling [25]. Similarly, ERK1/2 pathway activity is a key driver of melanoma growth and can coordinate transcriptional and metabolic programs that sustain survival under stress [37]. The present observation that co-treatment suppresses both p-AKT and p-ERK1/2 more strongly than single agents supports a “network inhibition” interpretation: simultaneous weakening of parallel survival branches may reduce the capacity of melanoma cells to compensate through pathway cross-talk, thereby lowering the threshold for energetic collapse and programmed cell death. This concept aligns with the broader rationale for combination interventions in cancer biology, where targeting multiple, partially redundant survival modules can generate synergy, while reducing escape routes [17,18]. The dual inhibition of PI3K/AKT and MAPK/ERK1/2 pathways observed here provides a rational basis for testing CUR/RSV in combination with existing melanoma therapies in vivo, including targeted inhibitors and immunotherapies.

The selectivity of pathway modulation is also informative. Normal melanocytes showed minimal changes in AKT/ERK1/2 phosphorylation and in apoptotic/necroptotic markers under the same exposures, consistent with preserved viability and ATP levels. This differential response may reflect lower basal dependence of normal melanocytes on hyperactivated survival signaling and lower proliferative/metabolic demand, providing a mechanistic basis for the therapeutic window observed. Such selectivity is particularly relevant for nutritional strategies, because tolerability is a key determinant of feasibility for long-term use, whether for prevention, risk reduction, or adjunctive therapy.

Physiological relevance and translational feasibility must, however, be carefully considered. Notably, the observed synergy suggests that effective biological responses may be achievable at lower concentrations than those required for single agents, an important consideration for in vivo dosing and formulation strategies. The concentrations of CUR and RSV used in vitro exceed plasma levels typically achieved after oral intake of native compounds, reflecting well-recognized limitations in bioavailability, rapid metabolism, and low systemic exposure, particularly for curcumin [38,39]. Accordingly, future in vivo studies should prioritize optimized delivery systems and localized administration approaches to recapitulate the multi-pathway disruption observed in vitro.

Curcumin bioavailability is widely recognized as a major limitation [38,39]. Formulation strategies (e.g., lipid-based systems, micelles, nanoparticles, phospholipid complexes) and localized delivery approaches may therefore be critical for translating CUR/RSV synergy into physiologically attainable exposure ranges [40,41,42]. In this context, the observed synergy has practical significance, as cooperative interactions may permit biological efficacy at lower effective concentrations than required for single agents, potentially narrowing the gap between in vitro activity and achievable exposure [17,18]. Moreover, topical or localized delivery strategies may be particularly relevant for cutaneous melanoma, where high local concentrations can be achieved while minimizing systemic exposure, and co-delivery platforms for CUR and RSV have been explored to enhance delivery and efficacy in melanoma-related models [43]. Future work should therefore evaluate whether optimized formulations and delivery routes can reproduce the multi-pathway disruption observed in vitro at physiologically attainable concentrations.

Several limitations warrant emphasis. First, the present study used one melanoma cell line and one normal melanocyte model. Given melanoma heterogeneity, validation across additional melanoma subtypes, mutational backgrounds, and therapy-resistant lines, as well as patient-derived systems and three-dimensional models, will be important to generalize the findings [20]. Second, although RIP and MLKL phosphorylation support necroptotic signaling, functional confirmation is needed to establish causality and quantify contribution, for example, through pharmacologic inhibition or genetic perturbation of RIPK/MLKL components, and through assessment of whether blocking necroptosis shifts death toward apoptosis, or restores viability [31,32]. Third, upstream metabolic determinants, such as mitochondrial membrane potential, ROS dynamics, glycolytic flux, and mitochondrial respiration, were not directly measured; incorporating these readouts would clarify whether ATP depletion represents an initiating metabolic trigger or a downstream consequence of signaling collapse and death execution. Fourth, long-term recovery or regrowth of melanoma cells after treatment withdrawal was not assessed, and such experiments would be informative to determine whether CUR/RSV induces durable growth suppression or allows adaptive escape. Finally, the potential interaction of CUR/RSV with established melanoma therapies, including MAPK-targeted inhibitors and immunotherapies, remains unexplored and should be addressed with rigorous combination designs and synergy evaluation frameworks [17,18,20].

**Figure 6:**
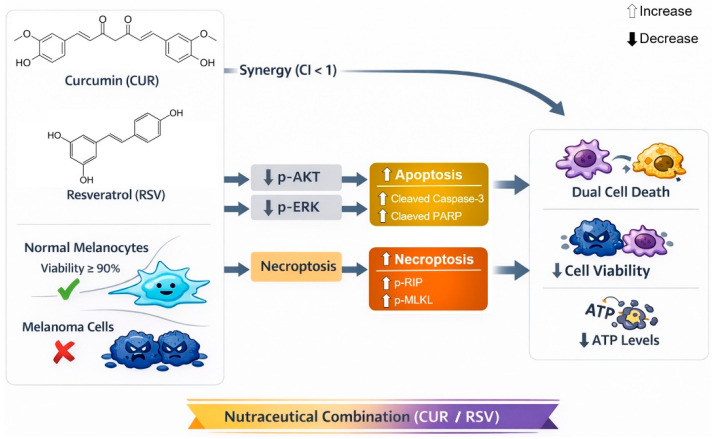
Proposed mechanism of CUR and RSV synergistic effects in melanoma cells. Co-treatment selectively reduces melanoma cell viability while sparing normal melanocytes. The combination suppresses pro-survival signaling via decreased AKT and ERK1/2 phosphorylation, activates apoptosis via caspase-3 and PARP cleavage, and induces necroptosis via increased phosphorylation of RIP and MLKL, resulting in ATP depletion and impaired cellular function.

Despite these limitations, the present results support a coherent mechanistic model: CUR/RSV selectively suppresses AKT/ERK1/2 survival signaling in melanoma cells, imposes metabolic stress with ATP depletion, enforces G0/G1 arrest, and engages complementary programmed cell death pathways, including apoptosis and necroptosis. Together, these findings provide a clear experimental and conceptual foundation for subsequent in vivo validation and preclinical development of CUR/RSV-based combination strategies, including their evaluation as adjunctive approaches alongside standard melanoma therapies in more complex preclinical models. Figure 6 provides a schematic summary integrating these pathways:

## 5. Conclusions

CUR and RSV co-treatment exerts synergistic and selective anticancer effects in melanoma G361 cells, characterized by reduced viability and ATP production, induction of G0/G1 arrest, and robust activation of apoptosis and necroptosis. Mechanistically, the combination suppresses AKT/ERK1/2 pro-survival signaling while increasing caspase-3/PARP cleavage and RIP/MLKL phosphorylation, indicating engagement of complementary programmed cell death programs. Under identical conditions, normal melanocytes exhibit comparatively limited metabolic and signaling perturbation, supporting a favorable selectivity profile. Future studies should validate these findings across additional melanoma subtypes and advanced models, incorporate functional pathway inhibition to confirm death mechanisms, and address bioavailability constraints through formulation optimization and in vivo pharmacokinetic-pharmacodynamic evaluation.

## Figures and Tables

**Table 1:** Primary antibodies used for Western blot analysis.

Target Protein	Molecular Weight (kDa)	Host Species	Company	Catalog No.	Dilution
Phospho-Akt	60	Rabbit	Cell Signaling Technology	#9271	1:500
Total Akt	60	Rabbit	Cell Signaling Technology	#9272	1:500
Phospho-ERK1/2	44, 42	Rabbit	Cell Signaling Technology	#4370	1:500
ERK1/2	44, 42	Rabbit	Cell Signaling Technology	#4695	1:500
Cleaved Caspase-3	17, 19	Rabbit	Cell Signaling Technology	#9664	1:500
Cleaved PARP	89	Rabbit	Cell Signaling Technology	#5625	1:500
Phospho-RIP	78	Rabbit	Cell Signaling Technology	#65746	1:500
RIP	78	Rabbit	Cell Signaling Technology	#3493	1:500
Phospho-MLKL	54	Rabbit	Cell Signaling Technology	#91689	1:500
MLKL	54	Rabbit	Cell Signaling Technology	#14993	1:500
Β-actin	42	Mouse	Sigma–Aldrich	A2228	1:1000

## Data Availability

The original contributions presented in the study are included in the article; further inquiries should be directed to the corresponding author.

## Supplementary Materials

The following supporting information can be downloaded at https://www.mdpi.com/article/10.3390/nu18030548/s1, Table S1: Summary of statistical analyses for all quantitative comparisons presented in the main figures.

## Abbreviations

The following abbreviations are used in this manuscript:

AKT	protein kinase B
ANOVA	analysis of variance
ATP	adenosine triphosphate
BCA	bicinchoninic acid
CI	combination index
CO_2_	carbon dioxide
CUR	curcumin
CTLA-4	cytotoxic T-lymphocyte–associated protein 4
DAMP	damage-associated molecular pattern
DAPI	4′,6-diamidino-2-phenylindole
DMEM	Dulbecco’s modified Eagle medium
DMSO	dimethyl sulfoxide
ERK	extracellular signal-regulated kinase
FBS	fetal bovine serum
G0/G1	gap 0/gap 1 phase
G2/M	gap 2/mitosis phase
HRP	horseradish peroxidase
IC_50_	half-maximal inhibitory concentration
MAPK	mitogen-activated protein kinase
MEK	mitogen-activated protein kinase kinase
MLKL	mixed lineage kinase domain-like protein
MTT	3-(4,5-dimethylthiazol-2-yl)-2,5-diphenyltetrazolium bromide
NF-κB	nuclear factor kappa B
PD-1	programmed cell death protein 1
PI3K	phosphoinositide 3-kinase
PVDF	polyvinylidene difluoride
RIP	receptor-interacting protein (kinase)
RIPA	radioimmunoprecipitation assay
RSV	resveratrol
SDS-PAGE	sodium dodecyl sulfate–polyacrylamide gel electrophoresis
SD	standard deviation
