# Supplementary material for: Dietary Polyphenols Curcumin and Resveratrol Exert Selective Anticancer Effects in Melanoma Cells
Source: Nutrients. 2026 Feb 6;18(3):548. doi: 10.3390/nu18030548 (PMC12899543; doi:10.3390/nu18030548)
Supplement: Supplementary file 1 [file nutrients-18-00548-s001.zip › Table S1. Summary of statistical analyses for all quantitative comparisons presented in the main figures..pdf]

| Figure | Cell line | Comparison | Summary | Adjusted p-value |
|---|---|---|---|---|
| Fig. 2B | HEMn-MP | control vs. CUR | **** | <0.0001 |
| Fig. 2B | HEMn-MP | control vs. RSV | **** | <0.0001 |
| Fig. 2B | HEMn-MP | control vs. CUR/RSV | **** | <0.0001 |
| Fig. 2B | HEMn-MP | CUR vs. RSV | ns | 0.9743 |
| Fig. 2B | HEMn-MP | CUR vs. CUR/RSV | **** | <0.0001 |
| Fig. 2B | HEMn-MP | RSV vs. CUR/RSV | **** | <0.0001 |
| Fig. 2B | G361 | control vs. CUR | **** | <0.0001 |
| Fig. 2B | G361 | control vs. RSV | **** | <0.0001 |
| Fig. 2B | G361 | control vs. CUR/RSV | **** | <0.0001 |
| Fig. 2B | G361 | CUR vs. RSV | ** | 0.0012 |
| Fig. 2B | G361 | CUR vs. CUR/RSV | **** | <0.0001 |
| Fig. 2B | G361 | RSV vs. CUR/RSV | **** | <0.0001 |

| Figure | Cell line | Comparison | Summary | Adjusted p-value |
|---|---|---|---|---|
| Fig. 2C | HEMn-MP | control vs. CUR | *** | 0.0005 |
| Fig. 2C | HEMn-MP | control vs. RSV | ** | 0.0019 |
| Fig. 2C | HEMn-MP | control vs. CUR/RSV | **** | <0.0001 |
| Fig. 2C | HEMn-MP | CUR vs. RSV | ns | 0.6491 |
| Fig. 2C | HEMn-MP | CUR vs. CUR/RSV | ** | 0.0049 |
| Fig. 2C | HEMn-MP | RSV vs. CUR/RSV | ** | 0.0012 |
| Fig. 2C | G361 | control vs. CUR | **** | <0.0001 |
| Fig. 2C | G361 | control vs. RSV | **** | <0.0001 |
| Fig. 2C | G361 | control vs. CUR/RSV | **** | <0.0001 |
| Fig. 2C | G361 | CUR vs. RSV | ns | 0.8673 |
| Fig. 2C | G361 | CUR vs. CUR/RSV | **** | <0.0001 |
| Fig. 2C | G361 | RSV vs. CUR/RSV | **** | <0.0001 |

| Figure | Cell line | | Comparison | Summary | Adjusted p-value |
|---|---|---|---|---|---|
| Fig. 4A | HEMn-MP | G0/G1 | control vs. CUR | ns | 0.3217 |
| Fig. 4A | HEMn-MP | | control vs. RSV | * | 0.0166 |
| Fig. 4A | HEMn-MP | | control vs. CUR/RSV | *** | 0.0004 |
| Fig. 4A | HEMn-MP | | CUR vs. RSV | ns | 0.5745 |
| Fig. 4A | HEMn-MP | | CUR vs. CUR/RSV | ns | 0.4568 |
| Fig. 4A | HEMn-MP | | RSV vs. CUR/RSV | ns | 0.6927 |
| Fig. 4A | HEMn-MP | S | control vs. CUR | ns | >0.9999 |
| Fig. 4A | HEMn-MP | | control vs. RSV | ns | >0.9999 |
| Fig. 4A | HEMn-MP | | control vs. CUR/RSV | ns | >0.9999 |
| Fig. 4A | HEMn-MP | | CUR vs. RSV | ns | >0.9999 |
| Fig. 4A | HEMn-MP | | CUR vs. CUR/RSV | ns | >0.9999 |
| Fig. 4A | HEMn-MP | | RSV vs. CUR/RSV | ns | >0.9999 |
| Fig. 4A | HEMn-MP | G2/M | control vs. CUR | ns | 0.4364 |
| Fig. 4A | HEMn-MP | | control vs. RSV | * | 0.0221 |
| Fig. 4A | HEMn-MP | | control vs. CUR/RSV | *** | 0.0007 |
| Fig. 4A | HEMn-MP | | CUR vs. RSV | ns | 0.9059 |
| Fig. 4A | HEMn-MP | | CUR vs. CUR/RSV | ns | 0.1559 |
| Fig. 4A | HEMn-MP | | RSV vs. CUR/RSV | ns | 0.9324 |

| **Figure** | **Cell line** | | **Comparison** | **Summary** | **Adjusted p-value** |
|---|---|---|---|---|---|
| Fig. 4A | G361 | G0/G1 | control vs. CUR | *** | 0.0003 |
| Fig. 4A | G361 | | control vs. RSV | *** | 0.0003 |
| Fig. 4A | G361 | | control vs. CUR/RSV | **** | <0.0001 |
| Fig. 4A | G361 | | CUR vs. RSV | ns | <0.0001 |
| Fig. 4A | G361 | | CUR vs. CUR/RSV | *** | <0.0001 |
| Fig. 4A | G361 | | RSV vs. CUR/RSV | *** | 0.0007 |
| Fig. 4A | G361 | S | control vs. CUR | ns | 0.9338 |
| Fig. 4A | G361 | | control vs. RSV | ns | 0.9987 |
| Fig. 4A | G361 | | control vs. CUR/RSV | ns | 0.2521 |
| Fig. 4A | G361 | | CUR vs. RSV | ns | >0.9999 |
| Fig. 4A | G361 | | CUR vs. CUR/RSV | ns | 0.9715 |
| Fig. 4A | G361 | | RSV vs. CUR/RSV | ns | 0.7555 |
| Fig. 4A | G361 | G2/M | control vs. CUR | * | 0.0177 |
| Fig. 4A | G361 | | control vs. RSV | ** | 0.0051 |
| Fig. 4A | G361 | | control vs. CUR/RSV | **** | <0.0001 |
| Fig. 4A | G361 | | CUR vs. RSV | ns | >0.9999 |
| Fig. 4A | G361 | | CUR vs. CUR/RSV | ** | 0.0049 |
| Fig. 4A | G361 | | RSV vs. CUR/RSV | * | 0.0170 |

Relative density(p-AKT/AKT)

| Figure | Cell line | Comparison | Summary | Adjusted p-value |
|---|---|---|---|---|
| Fig. 5D | HEMn-MP | control vs. CUR | ns | 0.9970 |
| Fig. 5D | HEMn-MP | control vs. RSV | ns | 0.9759 |
| Fig. 5D | HEMn-MP | control vs. CUR/RSV | ns | 0.9898 |
| Fig. 5D | HEMn-MP | CUR vs. RSV | ns | 0.9966 |
| Fig. 5D | HEMn-MP | CUR vs. CUR/RSV | ns | 0.9996 |
| Fig. 5D | HEMn-MP | RSV vs. CUR/RSV | ns | 0.9995 |
| Fig. 5D | G361 | control vs. CUR | **** | <0.0001 |
| Fig. 5D | G361 | control vs. RSV | **** | <0.0001 |
| Fig. 5D | G361 | control vs. CUR/RSV | **** | <0.0001 |
| Fig. 5D | G361 | CUR vs. RSV | *** | 0.0001 |
| Fig. 5D | G361 | CUR vs. CUR/RSV | **** | <0.0001 |
| Fig. 5D | G361 | RSV vs. CUR/RSV | *** | 0.0005 |

Relative density(p-ERK1/2/ERK1/2)

| Figure | Cell line | Comparison | Summary | Adjusted p-value |
|---|---|---|---|---|
| Fig. 5D | HEMn-MP | control vs. CUR | ns | 0.3299 |
| Fig. 5D | HEMn-MP | control vs. RSV | ns | 0.3532 |
| Fig. 5D | HEMn-MP | control vs. CUR/RSV | ns | 0.6069 |
| Fig. 5D | HEMn-MP | CUR vs. RSV | ns | >0.9999 |
| Fig. 5D | HEMn-MP | CUR vs. CUR/RSV | ns | 0.9416 |
| Fig. 5D | HEMn-MP | RSV vs. CUR/RSV | ns | 0.9562 |
| Fig. 5D | G361 | control vs. CUR | *** | 0.0003 |
| Fig. 5D | G361 | control vs. RSV | **** | <0.0001 |
| Fig. 5D | G361 | control vs. CUR/RSV | **** | <0.0001 |
| Fig. 5D | G361 | CUR vs. RSV | *** | 0.0003 |
| Fig. 5D | G361 | CUR vs. CUR/RSV | **** | <0.0001 |
| Fig. 5D | G361 | RSV vs. CUR/RSV | **** | <0.0001 |

Relative density(p-MLKL/MLKL)

| Figure | Cell line | Comparison | Summary | Adjusted p-value |
|---|---|---|---|---|
| Fig. 5D | HEMn-MP | control vs. CUR | ns | 0.9998 |
| Fig. 5D | HEMn-MP | control vs. RSV | ns | 0.5222 |
| Fig. 5D | HEMn-MP | control vs. CUR/RSV | ** | 0.0071 |
| Fig. 5D | HEMn-MP | CUR vs. RSV | ns | 0.5624 |
| Fig. 5D | HEMn-MP | CUR vs. CUR/RSV | ** | 0.0064 |
| Fig. 5D | HEMn-MP | RSV vs. CUR/RSV | ** | 0.0013 |
| Fig. 5D | G361 | control vs. CUR | **** | <0.0001 |
| Fig. 5D | G361 | control vs. RSV | **** | <0.0001 |
| Fig. 5D | G361 | control vs. CUR/RSV | **** | <0.0001 |
| Fig. 5D | G361 | CUR vs. RSV | * | 0.0200 |
| Fig. 5D | G361 | CUR vs. CUR/RSV | **** | <0.0001 |
| Fig. 5D | G361 | RSV vs. CUR/RSV | **** | <0.0001 |

Relative density(p-RIP/RIP)

| Figure | Cell line | Comparison | Summary | Adjusted p-value |
|---|---|---|---|---|
| Fig. 5D | HEMn-MP | control vs. CUR | ns | >0.9999 |
| Fig. 5D | HEMn-MP | control vs. RSV | ns | >0.9999 |
| Fig. 5D | HEMn-MP | control vs. CUR/RSV | * | 0.0418 |
| Fig. 5D | HEMn-MP | CUR vs. RSV | ns | >0.9999 |
| Fig. 5D | HEMn-MP | CUR vs. CUR/RSV | * | 0.0411 |
| Fig. 5D | HEMn-MP | RSV vs. CUR/RSV | * | 0.0383 |
| Fig. 5D | G361 | control vs. CUR | ** | 0.0045 |
| Fig. 5D | G361 | control vs. RSV | *** | 0.0004 |
| Fig. 5D | G361 | control vs. CUR/RSV | **** | <0.0001 |
| Fig. 5D | G361 | CUR vs. RSV | ns | 0.1593 |
| Fig. 5D | G361 | CUR vs. CUR/RSV | **** | <0.0001 |
| Fig. 5D | G361 | RSV vs. CUR/RSV | **** | <0.0001 |

Relative density(p-RIP/RIP)

| Figure | Cell line | Comparison | Summary | Adjusted p-value |
|---|---|---|---|---|
| Fig. 5D | HEMn-MP | control vs. CUR | ns | >0.9999 |
| Fig. 5D | HEMn-MP | control vs. RSV | ns | >0.9999 |
| Fig. 5D | HEMn-MP | control vs. CUR/RSV | * | 0.0418 |
| Fig. 5D | HEMn-MP | CUR vs. RSV | ns | >0.9999 |
| Fig. 5D | HEMn-MP | CUR vs. CUR/RSV | * | 0.0411 |
| Fig. 5D | HEMn-MP | RSV vs. CUR/RSV | * | 0.0383 |
| Fig. 5D | G361 | control vs. CUR | ** | 0.0045 |
| Fig. 5D | G361 | control vs. RSV | *** | 0.0004 |
| Fig. 5D | G361 | control vs. CUR/RSV | **** | <0.0001 |
| Fig. 5D | G361 | CUR vs. RSV | ns | 0.1593 |
| Fig. 5D | G361 | CUR vs. CUR/RSV | **** | <0.0001 |
| Fig. 5D | G361 | RSV vs. CUR/RSV | **** | <0.0001 |

Relative density(Cleaved Caspase-3/β-actin)

| Figure | Cell line | Comparison | Summary | Adjusted p-value |
|---|---|---|---|---|
| Fig. 5D | HEMn-MP | control vs. CUR | ns | 0.9976 |
| Fig. 5D | HEMn-MP | control vs. RSV | ns | 0.9610 |
| Fig. 5D | HEMn-MP | control vs. CUR/RSV | ns | 0.9818 |
| Fig. 5D | HEMn-MP | CUR vs. RSV | ns | 0.9902 |
| Fig. 5D | HEMn-MP | CUR vs. CUR/RSV | ns | 0.9976 |
| Fig. 5D | HEMn-MP | RSV vs. CUR/RSV | ns | 0.9994 |
| Fig. 5D | G361 | control vs. CUR | *** | 0.0004 |
| Fig. 5D | G361 | control vs. RSV | **** | <0.0001 |
| Fig. 5D | G361 | control vs. CUR/RSV | **** | <0.0001 |
| Fig. 5D | G361 | CUR vs. RSV | ** | 0.0089 |
| Fig. 5D | G361 | CUR vs. CUR/RSV | **** | <0.0001 |
| Fig. 5D | G361 | RSV vs. CUR/RSV | **** | <0.0001 |

Relative density(Cleaved PARP/β-actin)

| Figure | Cell line | Comparison | Summary | Adjusted p-value |
|---|---|---|---|---|
| Fig. 5D | HEMn-MP | control vs. CUR | ns | 0.9991 |
| Fig. 5D | HEMn-MP | control vs. RSV | ns | 0.7919 |
| Fig. 5D | HEMn-MP | control vs. CUR/RSV | ns | 0.9857 |
| Fig. 5D | HEMn-MP | CUR vs. RSV | ns | 0.8556 |
| Fig. 5D | HEMn-MP | CUR vs. CUR/RSV | ns | 0.9967 |
| Fig. 5D | HEMn-MP | RSV vs. CUR/RSV | ns | 0.9328 |
| Fig. 5D | G361 | control vs. CUR | **** | <0.0001 |
| Fig. 5D | G361 | control vs. RSV | **** | <0.0001 |
| Fig. 5D | G361 | control vs. CUR/RSV | **** | <0.0001 |
| Fig. 5D | G361 | CUR vs. RSV | ns | 0.1534 |
| Fig. 5D | G361 | CUR vs. CUR/RSV | **** | <0.0001 |
| Fig. 5D | G361 | RSV vs. CUR/RSV | **** | <0.0001 |
